# Yttrium-90 quantitative phantom study using digital photon counting PET

**DOI:** 10.1186/s40658-021-00402-6

**Published:** 2021-07-27

**Authors:** Joey Labour, Philippe Boissard, Thomas Baudier, Fouzi Khayi, David Kryza, Pascale Veyrat Durebex, Sandrine Parisse-Di Martino, Thomas Mognetti, David Sarrut, Jean-Noël Badel

**Affiliations:** 1 CREATIS; CNRS UMR 5220; INSERM U 1044; Université de Lyon; INSA-Lyon; Université Lyon 1, Lyon, France; 2 Centre de lutte contre le cancer Léon Bérard, Lyon, France; 3 Hospices Civils de Lyon; Université de Lyon; Université Claude Bernard Lyon 1; LAGEPP UMR 5007 CNRS, Lyon, France

**Keywords:** Radioembolisation, Digital photon counting, PET, Listmode reconstruction, Dosimetry, Monte Carlo simulation

## Abstract

**Background:**

PET imaging of ^90^Y-microsphere distribution following radioembolisation is challenging due to the count-starved statistics from the low branching ratio of *e*^+^/*e*^−^ pair production during ^90^Y decay. PET systems using silicon photo-multipliers have shown better ^90^Y image quality compared to conventional photo-multiplier tubes. The main goal of the present study was to evaluate reconstruction parameters for different phantom configurations and varying listmode acquisition lengths to improve quantitative accuracy in ^90^Y dosimetry, using digital photon counting PET/CT.

**Methods:**

Quantitative PET and dosimetry accuracy were evaluated using two uniform cylindrical phantoms specific for PET calibration validation. A third body phantom with a 9:1 hot sphere-to-background ratio was scanned at different activity concentrations of ^90^Y. Reconstructions were performed using OSEM algorithm with varying parameters. Time-of-flight and point-spread function modellings were included in all reconstructions. Absorbed dose calculations were carried out using voxel S-values convolution and were compared to reference Monte Carlo simulations. Dose-volume histograms and root-mean-square deviations were used to evaluate reconstruction parameter sets. Using listmode data, phantom and patient datasets were rebinned into various lengths of time to assess the influence of count statistics on the calculation of absorbed dose. Comparisons between the local energy deposition method and the absorbed dose calculations were performed.

**Results:**

Using a 2-mm full width at half maximum post-reconstruction Gaussian filter, the dosimetric accuracy was found to be similar to that found with no filter applied but also reduced noise. Larger filter sizes should not be used. An acquisition length of more than 10 min/bed reduces image noise but has no significant impact in the quantification of phantom or patient data for the digital photon counting PET. 3 iterations with 10 subsets were found suitable for large spheres whereas 1 iteration with 30 subsets could improve dosimetry for smaller spheres.

**Conclusion:**

The best choice of the combination of iterations and subsets depends on the size of the spheres. However, one should be careful on this choice, depending on the imaging conditions and setup. This study can be useful in this choice for future studies for more accurate ^90^Y post-dosimetry using a digital photon counting PET/CT.

## Background

Liver radioembolisation or selective internal radiation therapy (SIRT) is an intra-arterial method used in clinical practice to treat unresectable hepatic malignancies [1, 2]. Currently, SIRT can be performed either with ^90^Y or ^166^Ho microspheres. During ^90^Y-SIRT, the high energy *β*^−^ emitter ^90^Y particles which are encapsulated-in glass or labelled to resin microspheres are administered through selected branches of the hepatic artery which feed the tumours. This method ensures a regional biodistribution of the ^90^Y-microspheres delivering a highly localised absorbed dose to the perfused regions, sparing nearby organs at risk and healthy tissues with the advantage of a negligible radiation burden to both non-embolized portions and extra-hepatic tissues. The ^90^Y-SIRT method is widely used owing to its clinical efficacy and relative safety [1–5].

At present, the prediction of the biodistribution of ^90^Y-microspheres is generally performed using ^99*m*^Tc-labelled macro-aggregated albumin (MAA), prior to treatment. However, ^99*m*^Tc-MAA biodistribution does not always match with post-therapy ^90^Y-microspheres distribution [6–10] and an assessment of the radionuclide biodistribution must be performed following treatment either by single-photon emission computed tomography (SPECT) or positron emission tomography (PET). This assessment is mainly done to detect any possible extrahepatic deposition of microspheres and determine the intrahepatic microsphere distribution over the perfused tumorous and non-tumorous liver tissue.

^90^Y SPECT imaging exploits bremsstrahlung photons, with various published energy windows [11], and has been used for post-SIRT treatment evaluation [12]. However, SPECT suffers from scatter, low spatial resolution and challenging quantitative analysis. Alternatively, ^90^Y PET imaging exploits a minor positron decay [13–18]. In 2004, Nickles et al. [19] first exploited this property to show the distribution of the regional absorbed dose delivered by ^90^Y therapies using PET, although difficult and time-consuming due to the count-starved statistics for annihilation photons. Activity distribution assessment after ^90^Y-SIRT was proved feasible in 2010 by Lhommel et al. [20, 21] with the help of time-of-flight (ToF) information added on PET/CT systems. Other studies followed and showed that ToF PET compared to non-ToF PET provided improved recovery in reconstructed quantitative data [20–26], outperforming at the same time ^90^Y bremsstrahlung SPECT [12, 22]. In 2007, Selwyn et al. [17] verified the branching ratio related to e ^+^/e ^−^ pair production during ^90^Y decay to be (31.86 ±0.47) ×10^−6^, following de-excitation from the 0^+^ excited state of ^90^Zr. The latest published value was from Dryák and Šolc [18] in 2020, who measured the branching ratio to be (32.6 ±0.4) ×10^−6^.

The recent digital PET systems are equipped with silicon photo-multiplier (SiPM) technology that replaces conventional photo-multiplier tubes (PMT). They allow enhanced ToF capability and coincidence timing resolution owing to faster and more compact electronics [27, 28]. They demonstrate better performances for sensitivity, spatial resolution, count rates, and overall image quality [29–34].

Reviewing previous studies, assessments for ^90^Y imaging were performed largely using criteria based on NEMA guidelines [35] and by evaluating detectability for diagnostic purposes rather than dosimetry calculations. In 2013, Willowson et al. [36] and Carlier et al. [23] showed that with the help of ToF information, higher detectability was reached with a small number of Ordered Subsets Expectation Maximisation (OSEM) iterations on Siemens Biograph mCT systems. Few studies evaluated OSEM reconstruction parameters using absorbed dose calculation tools. In 2014, Pasciak et al. [37] based on previous findings [23, 36] found that an additional 4.5-mm full width at half maximum (FWHM) point-spread function (PSF) modelling improved accuracy in absorbed dose distributions using dose-volume histograms (DVHs). In 2018, Siman et al. [38] studied a GE D690 PET/CT and found that 3 iterations with 12 subsets with additional PSF modelling and a 5.2 mm FWHM post-reconstruction Gaussian filter size provided the least root-mean-square deviation (RMSD) between their experimental and reference DVH.

This study focuses on the use of a digital photon counting (DPC)-PET for ^90^Y quantification for dosimetry purposes following SIRT. We considered the fully digital Philips VEREOS PET SiPM system, with a 1:1 coupling between the lutetium–yttrium oxyorthosilicate (LYSO) scintillator crystals and the SiPMs [29], showing improved timing resolution and signal-to-noise ratio (SNR) compared to conventional PMT-PET [39]. Wright et al. showed that DPC-PET detection of annihilation photons following ^90^Y-SIRT is feasible, demonstrating concordant visualisation with improved ^90^Y-to-background contrast of microsphere distribution with the DPC-PET compared to SPECT and PMT-PET systems [40–42].

Previous studies [23, 36–38] evaluated OSEM reconstruction parameters for PMT-PET systems with ToF resolutions around 550 ps. Therefore, suggested parameters in literature might not be suitable for the DPC-PET with a ToF resolution around 300 ps [29, 30, 32], due to the dependence of the convergence of OSEM-based algorithms on the ToF information [39]. Moreover, implementations of OSEM-based algorithms differ within systems and the OSEM algorithm implemented in the DPC-PET is based on listmode data, instead of sinograms, and use spherically symmetric volume elements to model the image, instead of voxels [43].

In this study, we investigated the accuracy of ^90^Y DPC-PET by evaluating the effect of OSEM reconstruction parameters and acquisition duration on estimating the absorbed dose distribution based on DVHs [44], as proposed by Siman et al. [38].

## Materials and methods

In order to evaluate the accuracy of image-based absorbed dose estimations from ^90^Y DPC-PET/CT, 3 phantoms were selected and imaged using a range of parameters. Acquired PET images were used as input activity maps to compute the absorbed dose distributions and DVHs. Obtained image-based distributions were compared to reference absorbed dose distributions computed with Monte Carlo simulations and the impact of several parameters, including volumes of interest (VOIs), activity levels, reconstruction parameters and acquisition lengths, were evaluated. The following subsections describe (1) the phantoms, (2) the acquisition and reconstruction parameters, (3) the algorithms used to compute the absorbed dose, (4) the figures of merit and (5) the clinical application using several patient image datasets acquired on the same DPC-PET/CT following ^90^Y-SIRT treatment.

### Phantoms and activities

**Fig. 1:**
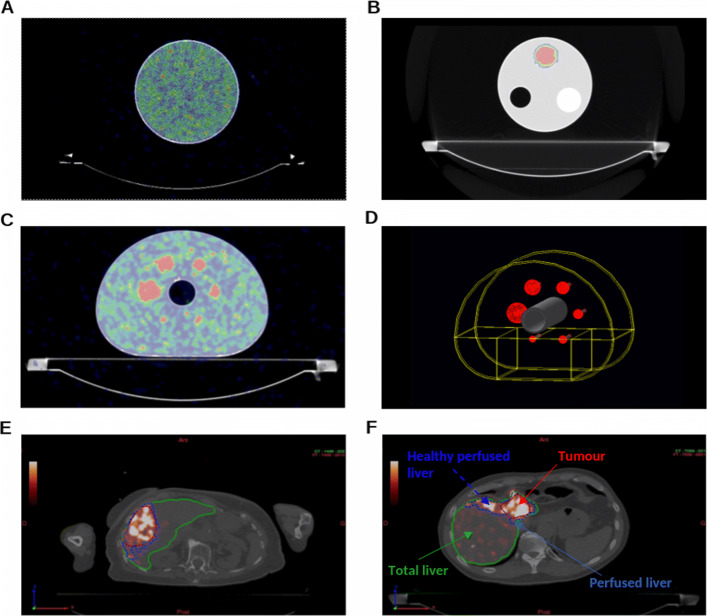
Axial slices of PET/CT images of **A** Ph1, **B** Ph2 and **C** Ph3. **D** Geometry of Ph3 modelled in GATE. **E**, **F** Examples of segmentation of liver VOIs for patients #2 and #3, respectively

A 6800-mL uniform cylindrical phantom (Ph1) (diameter ⌀ 21.6 cm; height h 18.6 cm) and a 5950-mL cylindrical phantom (⌀ 19.6 cm; h 19.7 cm) with a 300-mL cylindrical fillable insert (⌀ 4.5 cm; h 18.7 cm) (Ph2) were used for validation of quantitative recovered data following PET calibration for ^90^Y. All materials of Ph1 and Ph2 are made of PMMA. PET/CT fusion images of Ph1 and Ph2 are depicted in Fig. 1A and B, respectively.

A NEMA IEC body phantom (Ph3) in Fig. 1C was then used for quantitative measurements for dosimetry evaluations, consisting of a background compartment of approximately 9700 mL, a lung insert and an insert with six fillable spheres of diameters, 10, 13, 17, 22, 28 and 37 mm. The corresponding nominal volumes of the spheres ranged between 0.52 and 26.52 mL. The phantoms used in this study allow for quantitative activity recovery (or activity concentration recovery) and dosimetry evaluation in objects of different sizes.

Prior to phantom preparations, 100 *μ*L of diethylenetriaminepentaacetic acid (DTPA) with a concentration of 5 mg.mL ^−1^ was added to two vials, each containing 2850 MBq of ^90^YCl_3 in 1.03 mL. This was done to prevent the known effect of adsorption of ^90^YCl_3 on the inner PMMA walls of plastic phantoms which may negatively affect PET quantitative imaging studies [45]. Activities were measured using an Easypharma HE Lemer Pax activimeter calibrated for ^90^Y under national standards. Prepared syringes used for filling Ph1, Ph2 and Ph3 were flushed several times to transfer the maximum amount of activity into the phantom volumes. All syringes were also measured for residual activity to allow for the calculation of the net transferred activity.

**Cylindrical phantoms, Ph1 and Ph2.** Ph1 was filled with 2130 MBq of ^90^YCl_3 in water. The 300-mL water insert in Ph2 was filled with 540 MBq of ^90^YCl_3 and placed in a cold water background. The reference initial activity concentrations at injection (*AC*_*ref*,*init*_) were 0.31 MBq mL ^−1^ and 1.83 MBq mL ^−1^, which are the total net injected activity (*A*_*ref*,*init*_) in each region divided by the volume of the considered region, for Ph1 and the insert in Ph2, respectively.

**NEMA IEC body phantom, Ph3.** A stock solution was prepared for filling the spheres by combining 225 MBq of ^90^YCl_3 with 100 mL of water. An activity of 2355 MBq of ^90^YCl_3 was added to the 9700 mL water background. The *AC*_*ref*,*init*_ in the spheres and background compartment were 2.25 MBq mL ^−1^ and 0.24 MBq mL ^−1^ at injection, respectively. A sphere-to-background ratio (SBR) of 9:1 was obtained, similar to that in the QUEST study [45].

### Image acquisition

Image acquisitions for all phantoms were performed over six consecutive days (two half lives of ^90^Y) to analyse the response of the PET with decreasing activity concentrations. Markers were placed to allow for reproducible placement of the phantoms between daily scans. Data acquisitions were performed in *listmode* format. The acquisition lengths were 30 min per bed (min/bed) for both Ph1 and Ph2 and 15 min/bed for Ph3.

### Image reconstruction

All image reconstructions were performed with ToF information and using relaxed List Mode Ordered Subset Expectation Maximisation (LMOSEM) algorithm [43] implemented on Philips PET systems, with isotropic voxels of 2 ×2 ×2 mm^3^. They were post-treated with a regularised version of the Richardson-Lucy algorithm for resolution recovery [46, 47] with the default recommended parameters of the PSF modelling (1 iteration with a 6-mm regularisation kernel) which provide reasonable contrast recovery without noticeable Gibbs artefacts [48].

The listmode data for Ph1 and Ph2 were reconstructed with *Recon1*, the default clinical setup recommended by Philips; see Table 1. Several parameters were compared for Ph3, also listed in Table 1. The number of iterations were fixed to 1, 2 or 3 to limit image noise amplification. The number of subsets were varied with 10, 20 or 30 subsets to cover the range of suggested number of subsets used in previous studies [23, 36–38, 45, 49–62]. Post-reconstruction Gaussian filters of varying sizes were applied, between 0 (no filter) and 8 mm FWHM with increments of 2 mm. In total, 45 combinations for reconstructions were compared for Ph3. Reconstruction parameter sets suggested in the literature were also tested, *Recon2* [38] and *Recon3* [23, 36, 37]. Reconstruction parameters are denoted i3s5-2mm for example for 3 iterations with 5 subsets and a 2-mm FWHM post-reconstruction Gaussian filter, with implemented PSF and ToF modellings.

**Table 1:** Parameter sets used for listmode data reconstructions

Reconstruction	Iterations	Subsets	Gaussian filter	PSF	ToF
parameter set			(mm @ FWHM)		
Various*	1, 2 or 3	10, 20 or 30	0, 2, 4, 6 or 8	Yes	Yes
*Recon1*	3	5	2	Yes	Yes
*Recon2*	3	12	5.2	Yes	Yes
*Recon3*	1	21	5	Yes	Yes

^*^45 possible combinations of parameters for evaluation.

Finally, thanks to listmode data, datasets for Ph3 were rebinned into various acquisition lengths, from 5 to 15 min/bed, in order to evaluate the impact of the counts statistics on dosimetry and investigate if shorter acquisitions might be used.

### Absorbed dose computation

Monte Carlo simulations were used to estimate the reference absorbed dose distributions in the 3 phantoms and inserts therein, according to the known *AC*_*ref*,*init*_ in each region at injection. These reference absorbed dose distributions were compared to the ones that can be estimated from the ^90^Y PET reconstructed images. The image-based absorbed dose computations were performed first with the voxel S-values (VSV) kernel-based convolution method for the various reconstruction parameters applied (see Table 1). The local deposition method (LDM) was also used for comparison purposes.

**Reference absorbed dose.** Monte Carlo simulations were performed with the Geant4 Application for Tomographic Emission (GATE) platform 9.0 [63, 64] using GEANT4 10.5 [65]. The geometry, dimensions and material composition of each phantom were modelled. The modelled geometry for Ph3 is shown in Fig. 1D. The physics list named emstandard_opt4 was used^1^. It contains the GEANT4 most accurate standard and low-energy models for electromagnetic processes recommended for medical applications [66]. Range production cuts were set to 1 mm for electrons and photons in the whole geometry. In GEANT4, it means that secondary particles are only created and tracked when their expected range in the current material is larger than this distance. No variance reduction technique was used. The *β*^−^ radioactive sources of ^90^Y were simulated by homogeneous *generic ion sources* in each sphere and the background compartment. The absorbed doses were scored with 2×2×2 mm^3^ voxels sizes. The number of primary particles was adapted for each phantom region in a single simulation for an entire phantom according to the relative experimental *AC*_*ref*,*init*_ in each region, such as to reach a statistical Type-A uncertainty of lower than 1% on the estimated mean absorbed dose values. This corresponds for example to about 6×10^5^ primary generated particles for the smallest 10 mm sphere in Ph3. Final absorbed dose values were scaled according to the known accumulated activities in all injected regions.

**Image-based absorbed dose.** Absorbed dose distributions were first computed from the PET images with DOSIsoft® (Cachan, France) with the VSV dose kernel convolution algorithm following the MIRD formalism [67–69]. It is considered as a compromise between more simplified calculation models (such as the LDM multiplicative approach) and Monte Carlo calculations, allowing to achieve accurate absorbed dose distribution information in clinic [68–72]. Calculations were also performed using LDM for comparison purposes.

**Partition model.** Mean absorbed dose estimations were also carried out with the simplified MIRD formalism (*D*_*MIRD*_) for ^90^Y, using the partition model [73], according to:

1 $$  D_{MIRD} = \frac{A_{ref,init} (GBq)}{M (kg)} \times 49.67  $$

where *M* is the mass of each phantom region injected with *A*_*ref*,*init*_, respectively.

### Dosimetry-based figures of merit

For Ph1 and the filled insert in Ph2, VOIs were defined using the co-registered CT to PET images, using at first the exact complete internal dimensions of intended VOIs, denoted *VOI*_*outer*_, and secondly using reduced dimensions to avoid edge partial volume effects (PVE), denoted *VOI*_*inner*_. For Ph3, spherical VOIs were defined for the 6 spheres using the exact internal diameter of each sphere on the CT images. The DVH of each VOI was computed as suggested in [38].

#### DVH and RMSD

The reference Monte Carlo, image-based VSV convolution and image-based LDM DVHs are denoted $DVH_{ref}^{MC}, DVH_{pet}^{VSV}$ and $DVH_{pet}^{LDM}$, respectively. For each parameter set, *r*, used for image reconstruction and sphere size, ⌀, in Ph3, differences between the absorbed dose distributions using VSV convolution were evaluated by the RMSD between their respective $DVH_{ref,\diameter }^{MC}$ and $DVH_{pet,\diameter }^{VSV}$, see Eq. 2.

2 $$ RMSD_{\diameter,r} = \sqrt{ \frac{ \sum_{i=0}^{N-1}(DVH_{ref,\diameter,i}^{MC} - DVH_{pet,\diameter,r,i}^{VSV})^{2}}{N}}  $$

where N is the total number of points in which the absorbed dose-axes of the DVHs are sampled.

Comparisons using RMSD as in Eq. 2 were also performed between different DVHs obtained by varying acquisition lengths, e.g. between a 15- and a 10-min/bed acquisitions for Ph3.

#### *D*_*mean*_ and *D*_50*%*_

For all phantoms, comparisons between $DVH_{ref}^{MC}, DVH_{pet}^{LDM}$ and $DVH_{pet}^{VSV}$ were performed using differences in the mean absorbed doses, *D*_*mean*_. *D*_*mean*_ is denoted as $\overline {D}_{ref}^{MC}, \overline {D}_{pet}^{VSV}$ and $\overline {D}_{pet}^{LDM}$ for the reference Monte Carlo simulations, VSV convolution and LDM, respectively. Similar comparisons were made using the absorbed doses at 50% volume, *D*_50*%*_, denoted $D_{ref,50\%}^{MC}, D_{pet,50\%}^{VSV}$ and $D_{pet,50\%}^{LDM}$ for each corresponding calculation method.

#### *RC*_*AC*_ and *RC*_*Dose*_

In addition, instead of the NEMA contrast recovery coefficient (CRC) definition [35] that aims at lesion detection rather than absorbed dose estimation, we used the mean activity concentration recovery coefficient (*RC*_*AC*_) and the mean absorbed dose recovery coefficient (*RC*_*Dose*_) using VSV convolution for quantitative analysis with decreasing activity concentrations, see Eqs. 3 and 4.

3 $$\begin{array}{@{}rcl@{}} \textrm{\(RC_{AC,\diameter}\)} & = & \frac{AC_{pet,\diameter}}{AC_{ref,\diameter}}  \end{array} $$

4 $$\begin{array}{@{}rcl@{}} \textrm{\(RC_{Dose,\diameter}\)} & = & \frac{\overline{D}_{pet,\diameter}^{VSV}}{\overline{D}_{ref,\diameter}^{MC}}  \end{array} $$

where for each ⌀,*AC*_*pet*,⌀_ is the mean activity concentration measured from reconstructed PET images and *AC*_*ref*,⌀_ is the reference activity concentration at the start of each acquisition.

### Clinical application

The dosimetric impact of reducing PET acquisition duration was investigated on five patients treated by ^90^Y-SIRT in the local hospital; see Table 2. The initial acquisition length was 15 min/bed position. Listmode datasets were used to artificially decrease the acquisition length down to 10 and 5 min/bed position during the reconstruction step. Each patient’s listmode data was reconstructed using the reconstruction parameters chosen following the evaluation using DVH and RMSD on Ph3 (see results in “Comparison using RMSD” section, paragraph *Choice of reconstruction parameter set*).

**Table 2:** Patient characteristics

Patient	Sex,	Tumour	Microsphere	Injected ^90^Y	Treatment	WLV-PLV-TV
	Age	type	material	activity (GBq)	approach	(cm^3^-cm^3^-cm^3^)
#1	M, 65	HCC	Glass	2.463	Lobar	2470-635-365
#2	F, 92	mCRC	Resin	0.716	Segmental	870-330-380
#3	M, 67	mCRC	Resin	0.800	Lobar	1010-127-50
#4	F, 16	FLC	Resin	1.752	Whole liver	2900-1020-70
#5	F, 67	mCRC	Resin	1.479	Whole liver	4240-2610-230

*HCC* hepatocellular carcinoma, *FLC* fibrolamellar carcinoma, *mCRC* hepatic metastases from colorectal cancer

For all considered patients, different VOIs were delineated by an experienced clinician following the local hospital protocol, including (1) whole liver volume (WLV), (2) perfused liver volume (PLV), (3) tumour volume (TV) and (4) perfused normal liver volume (PNLV). 3D segmentations were performed using the DOSIsoft® software and the registered CT and PET images. The WLV and TV were manually segmented using the CT images. Only the largest visible lesion on the CT was selected per patient as the TV for illustration in this study. The PLV was delineated using a threshold of 5% of the maximum activity in the liver on the PET images. The PNLV was considered as the subtraction of the TV from the PLV.

Figure 1E and F depict axial slices of liver VOI segmentations for patients #2 and #3, respectively. DVH analysis was performed on the different VOIs, using the VSV convolution and LDM algorithms implemented in DOSIsoft®. The metrics used for comparison are the *D*_*mean*_ and *D*_50*%*_ as in phantoms, as well as the absorbed doses at 2% volume, *D*_2*%*_, denoted $D_{pet,2\%}^{VSV}$ and $D_{pet,2\%}^{LDM}$ for VSV convolution and LDM, respectively.

## Results

### Cylindrical phantoms Ph1 and Ph2

**Fig. 2:**
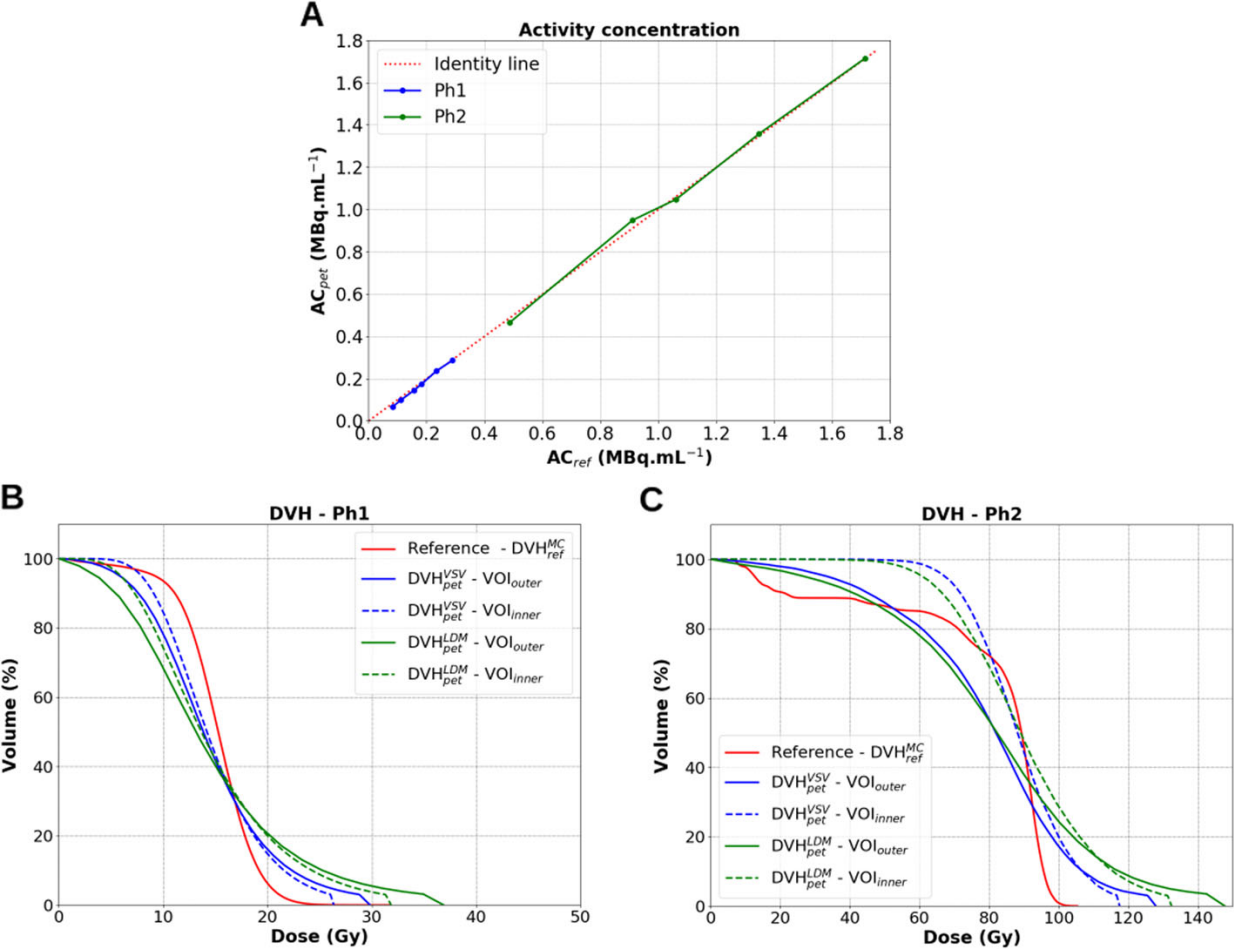
Quantitative accuracy of ^90^Y acquisitions for image reconstructions with *Recon1*. **A** *AC*_*pet*_ against *AC*_*ref*_ for both Ph1 and Ph2. **B**, **C** Comparisons of $DVH_{pet}^{VSV}$ (blue lines) and $DVH_{pet}^{LDM}$ (green lines) to $DVH_{ref}^{MC}$ (red lines) for Ph1 at *AC*_*ref*_ = 0.29 MBq mL ^−1^ and Ph2 at *AC*_*ref*_ = 1.71 MBq mL ^−1^, respectively

The first test was a sanity check to evaluate the PET response. Figure 2A depicts the measured mean activity concentrations from reconstructed PET images, *AC*_*pet*_, versus *AC*_*ref*_, for both Ph1 and Ph2 using *VOI*_*outer*_ (the exact internal dimensions of the VOI). *AC*_*ref*_ ranged from 0.08 to 0.29 MBq mL ^−1^ for Ph1, and from 0.49 to 1.71 MBq mL ^−1^ for Ph2.

On the first imaging day (highest *AC*_*ref*_), relative percentage differences between *AC*_*pet*_ and *AC*_*ref*_ were −1.1*%* and +0.1*%* for Ph1 at 0.29 MBq mL ^−1^ and Ph2 at 1.71 MBq mL ^−1^, respectively. The maximum relative percentage differences obtained over the range of activity concentrations studied were −19.6*%* and −4.5*%* for Ph1 at 0.08 MBq mL ^−1^ and Ph2 at 0.49 MBq mL ^−1^, corresponding to an absolute difference of −0.02 MBq mL ^−1^ for both Ph1 and Ph2 at the reported *AC*_*ref*_, respectively.

Figure 2B and C depict the calculated DVHs for Ph1 and Ph2, respectively, according to the computation methods used: $DVH_{ref}^{MC}$ (reference absorbed dose), $DVH_{pet}^{VSV}$ and $DVH_{pet}^{LDM}$ (PET image-based absorbed doses). The obtained DVHs illustrate the loss of accuracy brought by the use of images in the calculation of absorbed dose distributions. DVHs calculated with *VOI*_*outer*_ regions suffer from edge PVE effects compared to the ones based on *VOI*_*inner*_ regions for the known geometries. LDM compared to VSV convolution seems to favour the amplification of the maximum absorbed dose (*D*_*max*_) to the detriment of intermediate ones. Table 3 provides the *D*_*mean*_ and *D*_50*%*_ for both phantoms computed with each absorbed dose calculation method. Their percentage differences to the reference Monte Carlo simulations are also provided.

**Table 3:** Comparison of absorbed dose calculation methods through the *D*_*mean*_ and *D*_50*%*_ for Ph1 at 0.29 MBq mL ^−1^ and Ph2 at 1.71 MBq mL ^−1^, for both *VOI*_*outer*_ and *VOI*_*inner*_. Reconstructions were performed using *Recon1*

Calculation	Ph1 _*VOI*,*inner*_		Ph1 _*VOI*,*outer*_		Ph2 _*VOI*,*inner*_		Ph1 _*VOI*,*outer*_	
method	*D*_*mean*_	*D*_50*%*_	*D*_*mean*_	*D*_50*%*_	*D*_*mean*_	*D*_50*%*_	*D*_*mean*_	*D*_50*%*_
Ref. MC (Gy)	15.1	15.2	15.1	15.2	83.7	89.2	83.7	89.2
VSV conv. (Gy)	14.9	14.2	14.7	13.9	88.8	88.1	79.9	81.9
LDM (Gy)	15.0	13.7	14.8	13.1	90.5	89.0	82.7	82.2
Percent diff. (%)	−1.3	−6.6	−2.6	−8.6	+ 6.1	−1.3	−4.5	−8.2
MC vs VSV								
Percent diff. (%)	−0.7	−9.9	−2.6	−13.8	+ 8.1	−0.2	−1.1	−7.8
MC vs LDM								

### NEMA IEC body phantom Ph3

In this section, we proceed in the comparison of different reconstruction parameters for Ph3 using $DVH_{pet}^{VSV}$ and $DVH_{ref}^{MC}$ and their RMSD at the imaging point where *AC*_*ref*_ was equal to 2.18 MBq mL ^−1^ (*AC*_*ref*,*init*_ was 2.25 MBq mL ^−1^). We also evaluate the effect of acquisition length on absorbed dose distributions. The response of the PET is then evaluated using the *RC*_*AC*_ and *RC*_*Dose*_ for different *AC*_*ref*_ over two ^90^Y radioactive periods following phantom preparation. Comparisons using $DVH_{pet}^{LDM}$ are also reported.

#### Evaluation using $DVH_{pet}^{VSV}$

**Fig. 3:**
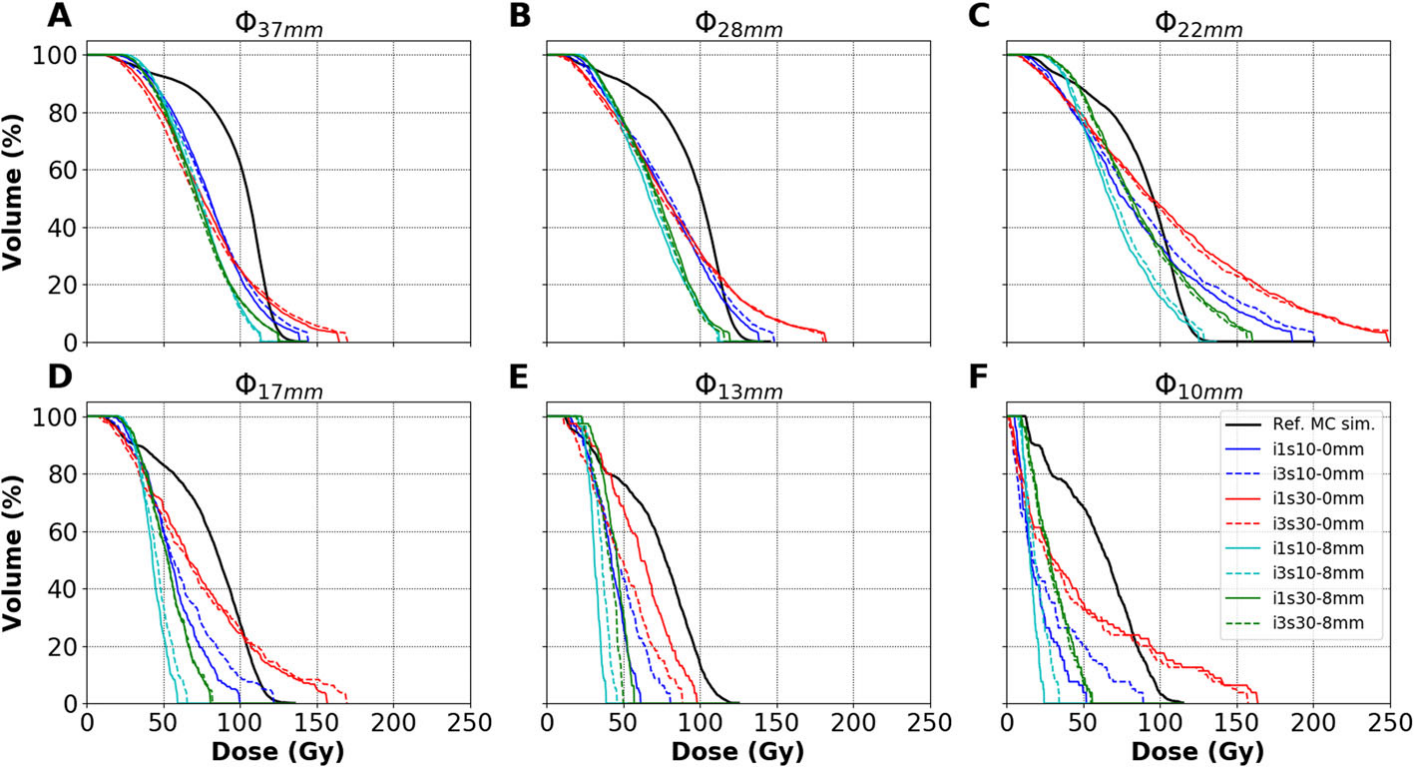
The $DVH_{pet}^{VSV}$ for 8 different reconstructions are compared to the $DVH_{ref}^{MC}$ (black curves) for each sphere of Ph3. Sphere sizes are represented in **A** 37 mm, **B** 28 mm, **C** 22 mm, **D** 17 mm, **E** 13 mm and **F** 10 mm. The dose-axis and volume-axis in each figure have the same corresponding limits

In total, 270 image-based $DVH_{pet}^{VSV}$ (45 reconstruction parameter sets described in “Image reconstruction” section for the 6 spheres) have been computed, and 6 reference $DVH_{ref}^{MC}$, corresponding to each sphere, have been simulated. For each sphere and each reconstruction, the $DVH_{pet}^{VSV}$ has been compared to the $DVH_{ref}^{MC}$. Figure 3 depicts the simulated $DVH_{ref}^{MC}$ (black curves) for each sphere and the $DVH_{pet}^{VSV}$ for 8 reconstructions per sphere (only extremes are depicted: 1 and 3 iterations, 10 and 30 subsets, 0 and 8 mm FWHM filter sizes).

**Varying post-reconstruction Gaussian filter.** As expected for all spheres, increasing the filter size reduced the *D*_*max*_ of the $DVH_{pet}^{VSV}$, e.g. between i1s30-0mm and i1s30-8mm, as seen in Fig. 3. It could be observed that too large a filter could not be suitable for dosimetry, specially with decreasing sphere sizes where the area under the curve can be significantly reduced (comparing cyan and green $DVH_{pet}^{VSV}$ in Fig. 3D–F).

**Varying subsets.** As expected, increasing the number of subsets led to an increase in the *D*_*max*_ of the $DVH_{pet}^{VSV}$ for all spheres, e.g. between i3s10-0mm and i3s30-0mm, as seen in Fig. 3. For the largest 28 and 37 mm spheres, 30 iterations compared to 10 iterations favoured noise amplification to the detriment of intermediate absorbed doses (comparing red and blue $DVH_{pet}^{VSV}$).

**Varying iterations.** The relationship in varying the number of iterations was less clear and intuitive than with the number of post-reconstruction filter or subsets. For spheres >20 mm (22, 28 and 37 mm), increasing the number of iterations did not incur significant change in the shape of the $DVH_{pet}^{VSV}$ or resulted in a slight increase in the *D*_*max*_, e.g. between i1s10-0mm and i3s10-0mm, as shown on Fig. 3. Increasing iterations from 1 to 3 did not seem to favour noise amplification for the largest spheres. On the other hand, more variations were observed for spheres <20 mm (10, 13, 17 mm) using the same comparison, e.g. between i1s10-0mm and i3s10-0mm.

**Equivalent updates.** Equivalent number of updates (product of the number of iterations and subsets) did not provide the same accuracy in $DVH_{pet}^{VSV}$ as would be expected, e.g. between i1s30-0mm and i3s10-0mm or between i1s30-8mm and i3s10-8mm.

#### Comparison using RMSD

**Fig. 4:**
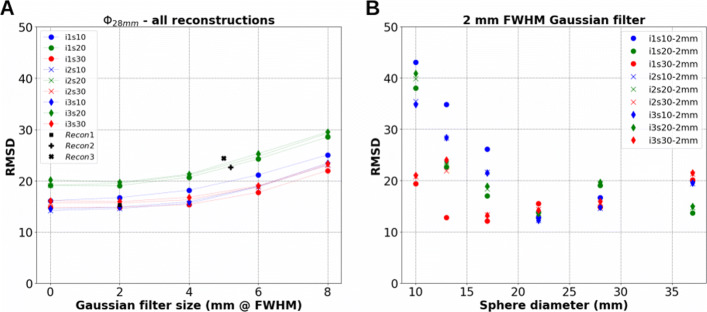
RMSD between $DVH_{pet}^{VSV}$ and $DVH_{ref}^{MC}$. **A** RMSD for all reconstructions for the 28-mm sphere against filter size. **B** RMSD against sphere sizes for reconstructions using a 2-mm FWHM post-reconstruction Gaussian filter only

Figure 4A outlines the RMSD between $DVH_{pet}^{VSV}$ and $DVH_{ref}^{MC}$ for the 28-mm sphere as an example, corresponding to 45 reconstructions (9 combinations of iterations and subsets each with 5 filter sizes). The figure also shows three additional RMSD values for *Recon1-3* in Table 1. Independent of the combination of iterations and subsets, the RMSD between $DVH_{pet}^{VSV}$ and $DVH_{ref}^{MC}$ were smallest when no (0 mm), or a 2-mm FWHM post-reconstruction Gaussian filter was applied. Similar observations were made for all spheres, except for the 22-mm sphere where the D _*max*_ could be amplified when no filter was applied, and agreed with the observations made in Fig. 3 when increasing the filter size.

From the previous findings, we now consider the use of a post-reconstruction Gaussian filter size of 2-mm FWHM for dosimetry, which can reduce noise in the reconstructed image while keeping the same accuracy as when no filter is applied. Figure 4B depicts the RMSD for all spheres obtained for reconstructions with a 2 mm FWHM filter only. Larger variations in RMSD were found for spheres <20 mm than spheres >20 mm. For the 10- to 17-mm spheres, i1s30-2mm provided the smallest RMSD. For the 22- to 37-mm spheres, the smallest RMSD were obtained using two combinations: i3s10-2mm for both the 22- and 28-mm spheres, and i1s20-2mm for the 37-mm sphere.

**Choice of reconstruction parameter set.**The reconstruction parameter set i3s10-2mm, as depicted by Fig. 3, provides a good compromise in reducing D _*max*_ and provides more accurate intermediate absorbed doses (*D*_20*%*_−*D*_80*%*_) for the larger spheres (22-37 mm). It also shows relatively low RMSDs in Fig. 4 for these spheres. This parameter set is therefore selected for image reconstructions in the following sections.

#### Effect of acquisition duration

**Fig. 5:**
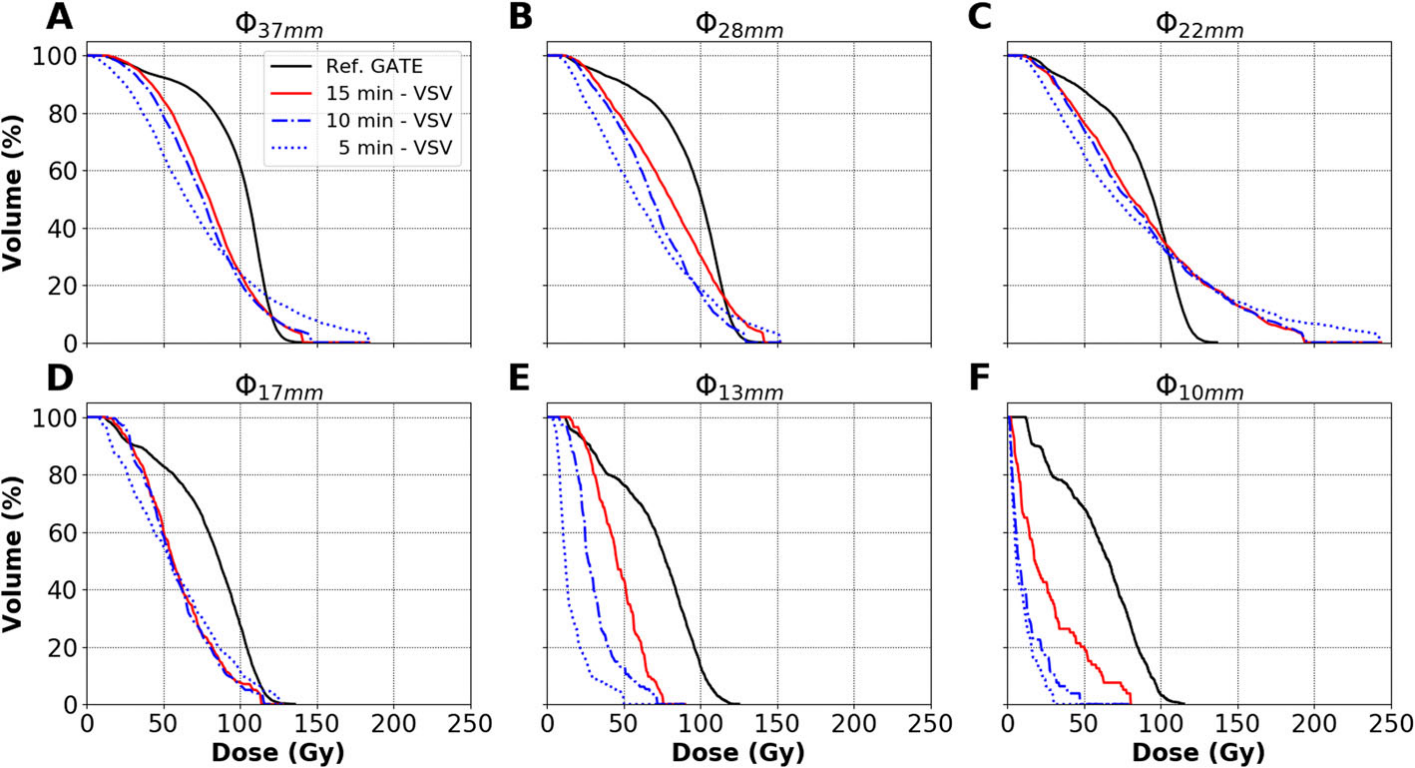
Effect of acquisition duration on $DVH_{pet}^{VSV}$ for all spheres. **A** 37 mm, **B** 28 mm, **C** 22 mm, **D** 17 mm, **E** 13 mm and **F** 10 mm. Reconstructions were performed using i3s10-2mm. The x-axis and y-axis in each figure have the same corresponding limits

Figure 5 depicts the effect of the acquisition duration on the $DVH_{pet}^{VSV}$, for the 6 spheres of Ph3. The RMSDs when comparing a 15 to a 10 min/bed acquisitions $DVH_{pet}^{VSV}$ were 3.2, 8.0, 1.6, 1.8, 23.2 and 19.4 for the 37- to 10-mm spheres, respectively. These corresponding RMSDs increased to 10.1, 12.7, 5.6, 6.8, 43.6 and 24.2, when comparing a 15 to a 5 min/bed $DVH_{pet}^{VSV}$, respectively.

#### *RC*_*AC*_ and *RC*_*Dose*_

**Fig. 6:**
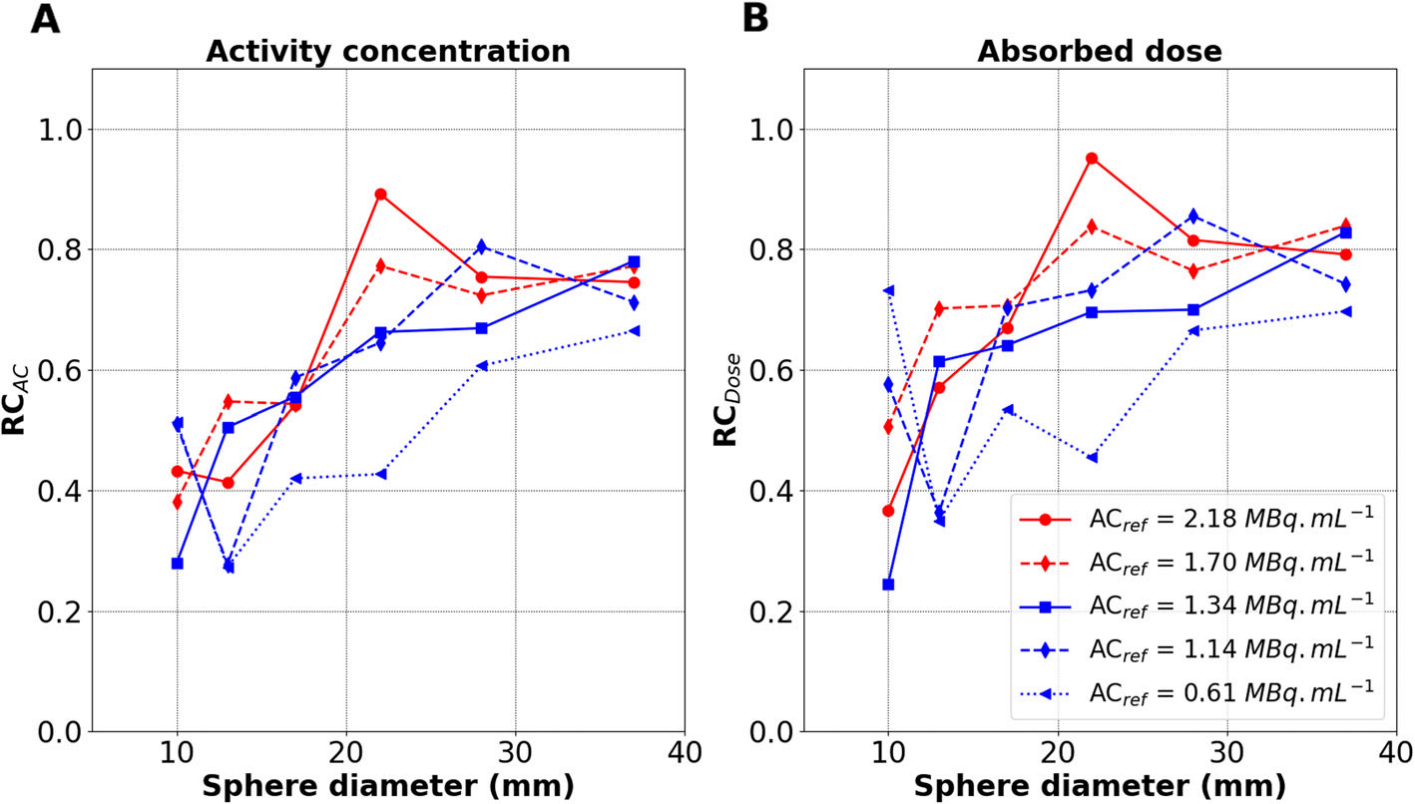
Recovery coefficients against sphere diameter for varying *AC*_*ref*_ over 6 days. **A** *RC*_*AC*_. **B** *RC*_*Dose*_. Reconstructions were performed using i3s10-2mm

Figure 6A and B depict the *RC*_*AC*_ and *RC*_*Dose*_ using VSV convolution (see Eqs. 3 and 4) for all the spheres with decreasing *AC*_*ref*_, respectively. *AC*_*ref*_ ranged between 0.61 and 2.18 MBq mL ^−1^. The activity concentration recovery performance is influenced by the count statistics related to the total activity present in the PET’s FOV. *RC*_*AC*_ and *RC*_*Dose*_ both decrease with decreasing *AC*_*ref*_ in the spheres. Overall, *RC*_*AC*_ and *RC*_*Dose*_ were comparable for all spheres and *AC*_*ref*_. *RC*_*Dose*_ was slightly greater than *RC*_*AC*_ for most of the considered object sizes as visible by comparison of Fig. 6A to B.

#### Comparison to LDM

Table 4 provides a comparison for Ph3 between the *D*_*mean*_ obtained with different calculation methods ($D_{MIRD}, \overline {D}_{ref}^{MC}, \overline {D}_{pet}^{VSV}$ and $\overline {D}_{pet}^{LDM}$, see the “Absorbed dose computation” and “Dosimetry-based figures of merit” sections) and their corresponding *D*_50*%*_, for the image acquisition where *AC*_*ref*_ was largest (2.18 MBq mL ^−1^). As expected, $\overline {D}_{ref}^{MC}$ decreased with decreasing sphere sizes since the sphere surface-to-volume ratio increases, leading to more absorbed dose delocalisation due to electrons exiting the spherical VOI. Moreover, $\overline {D}_{pet}^{VSV}$ dropped when estimated from the PET image compared to $\overline {D}_{ref}^{MC}$, e.g. from 89.5 to 59.8 Gy for the 17-mm sphere. *D*_*mean*_ and *D*_50*%*_ were comparable for each calculation method. Overall, *D*_*mean*_ values estimated by LDM were closer than VSV convolution to Monte Carlo simulated values.

**Table 4:** Comparison between absorbed dose estimations for all spheres (⌀_*imm*_). Reconstructions were performed using i3s10-2mm. All values in the table are in Gy

**VOI**	***D***_***MIRD***_	Ref. Monte Carlo		VSV convolution		LDM	
		${\overline {D}_{ref}^{MC}}$	${D_{ref,50\%}^{MC}}$	${\overline {D}_{pet}^{VSV}}$	${D_{pet,50\%}^{VSV}}$	$\overline {D}_{pet}^{LDM}$	${D_{pet,50\%}^{LDM}}$
⌀_10_***mm***	112	73.3	66.1	26.8	17.3	31.3	12.6
⌀_13_***mm***	112	81.1	77.4	46.3	45.3	51.2	48.7
⌀_17_***mm***	112	89.5	86.8	59.8	55.9	66.3	58.0
⌀_22_***mm***	112	93.8	95.2	89.2	81.0	96.4	84.8
⌀_28_***mm***	112	97.8	101.1	79.7	80.5	84.7	81.1
⌀_37_***mm***	112	101.8	105.8	80.5	80.0	83.7	78.5

### Clinical application

#### Reducing acquisition duration

**Fig. 7:**
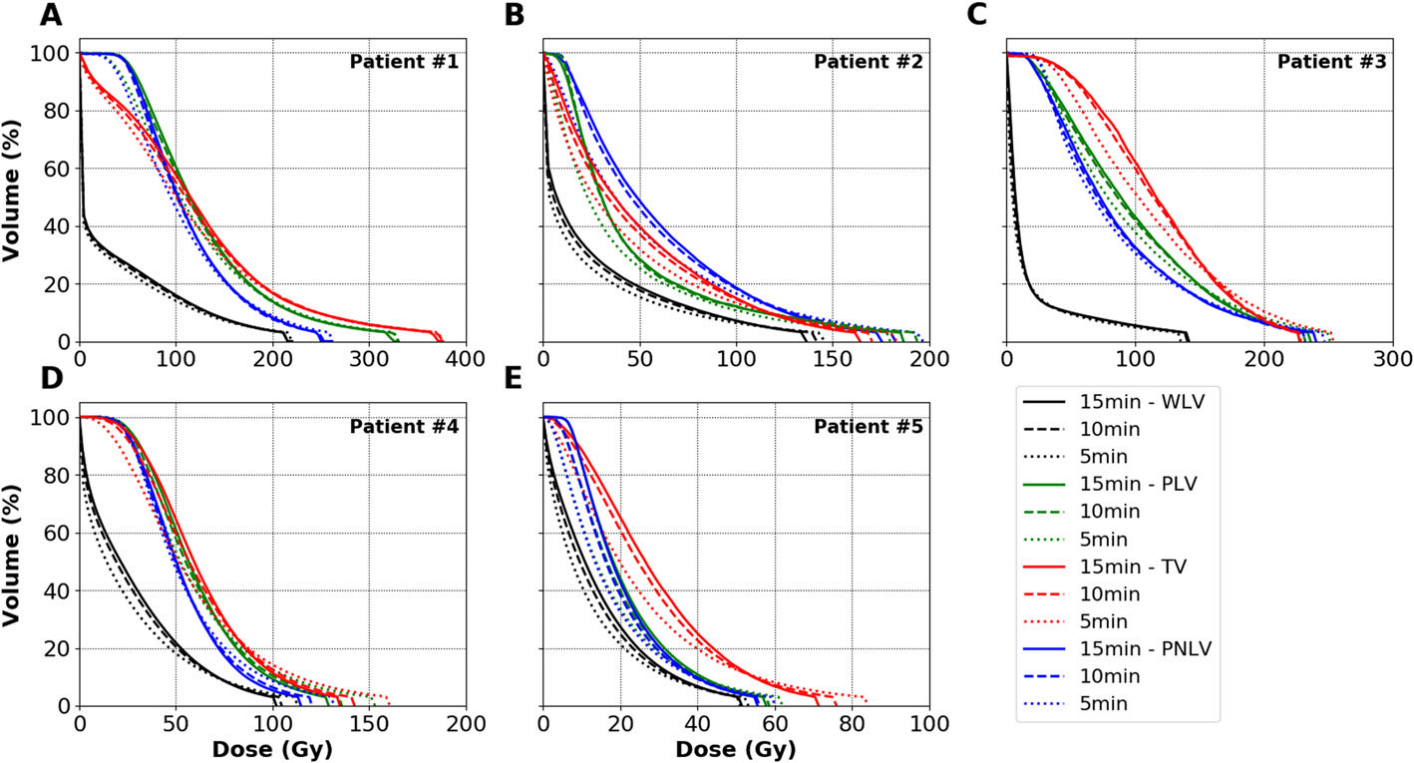
Effect of acquisition duration on $DVH_{pet}^{VSV}$ for 5 patients. Reconstructions were performed using i3s10-2mm. Volume-axes in all figures have the same corresponding limits

Figure 7 shows the influence of acquisition duration on the absorbed dose distributions for post ^90^Y-SIRT patient acquisitions, using i3s10-2mm reconstruction parameters, as previously recommended. Only minor differences were observed between $DVH_{pet}^{VSV}$ calculated for 10 and 15 min/bed acquisitions, for each of the liver VOIs delineated for all patients. The RMSDs between 10 and 15 min/bed acquisitions for patient #1 were 0.3, 1.2, 1.5 and 0.9 for the WLV, PLV, TV and PNLV, respectively. The RMSDs for each VOI between 5 and 15 min/bed acquisitions for patient #1 increased to 2.8, 18.3, 12.4 and 24.1 for the WLV, PLV, TV and PNLV, respectively.

Due to the size of the liver, the local protocol for the post ^90^Y-SIRT patients generally includes a two-bed, 15 min/bed acquisition. The reduction of acquisition duration on the specific patients studied here appeared to have no significant impact on dosimetry via $DVH_{pet}^{VSV}$. Also, the visual interpretation made by physicians were similar when comparing 10 to a 15 min/bed patient acquisitions. Therefore, we suggest a reduction of the acquisition duration from 15 to 10 min/bed, resulting in a total of 20 minutes instead of 30 minutes for 2 bed positions, helping to improve patient comfort.

#### Comparison to LDM

Table 5 provides subsequent results for patients #1 and #2 for comparisons between VSV convolution and LDM. *D*_*mean*_,*D*_50*%*_ and *D*_2*%*_ values are reported. LDM resulted in a systematically higher *D*_2*%*_ than VSV convolution for all VOIs, e.g. 247.9 Gy for LDM to 214.1 Gy for VSV convolution. *D*_*mean*_ and *D*_50*%*_ between the two calculation methods were comparable for all VOIs for all patients.

**Table 5:** Comparison of LDM to VSV convolution absorbed dose calculation methods through *D*_*mean*_,*D*_50*%*_ and *D*_2*%*_ for patients #1 and #2 and different VOIs. Reconstructions were performed using i3s10-2mm

**Patient, VOI**	VSV convolution			LDM		
	$\overline {D}^{VSV}_{pet}$	$D^{VSV}_{pet,50\%}$	$D^{VSV}_{pet,2\%}$	$\overline {D}^{LDM}_{pet}$	$D^{LDM}_{pet,50\%}$	$D^{LDM}_{pet,2\%}$
#1, WLV	38.1	1.3	242.7	38.5	0.9	263.4
#1, PLV	131.0	112.2	365.7	133.8	111.2	401.4
#1, TV	126.4	109.8	417.1	127.8	104.5	459.5
#1, PNLV	113.9	101.2	270.7	116.9	101.3	305.7
#2, WLV	25.1	5.4	157.8	25.5	3.6	178.1
#2, PLV	61.0	45.4	197.7	62.6	43.7	231.9
#2, TV	49.5	33.4	183.8	50.4	30.0	215.8
#2, PNLV	48.5	30.0	214.1	51.8	30.6	247.9

## Discussion

**Context.** The main goal of this work was to evaluate the influence of the acquired count statistics (acquisition length), phantom configuration and reconstruction parameters using a DPC-PET to improve quantitative accuracy in ^90^Y dosimetry for PET imaging. An initial check was performed using two cylindrical uniform phantoms. A third NEMA IEC body phantom was used to evaluate the relaxed LMOSEM algorithm parameters implemented in Philips reconstruction platforms for PET imaging. OSEM parameters were varied to find combinations of iterations, subsets and post-reconstruction Gaussian filter sizes which would provide the least difference between the $DVH_{ref}^{MC}$ using GATE and $DVH_{pet}^{VSV}$ using DOSIsoft® (Cachan, France). ToF and PSF modellings were considered in all reconstructions. The acquisition duration was varied by rebinning listmode phantom and patient datasets to determine the shortest acquisition duration that maintains an acceptable accuracy. Using the method suggested by Siman et al., the RMSD between $DVH_{ref}^{MC}$ and $DVH_{pet}^{VSV}$ was used to compare absorbed doses obtained with different datasets and dosimetry methodologies. Analysis using $DVH_{pet}^{LDM}$ was also performed, using specific reconstruction parameters following evaluation. No special intent was made toward improving image quality using NEMA standards [35] since the goal was to improve dosimetry accuracy.

**Ph1 and Ph2.** Considering mean activity concentrations and mean absorbed doses for Ph1 (at *AC*_*ref*_ equal to 0.29 MBq mL ^−1^) and Ph2 (at *AC*_*ref*_ equal to 1.71 MBq mL ^−1^), the DPC-PET was shown to produce accurate results (<5% using *VOI*_*outer*_) with large phantoms. Relative percentage differences between *AC*_*pet*_ and *AC*_*ref*_ using *VOI*_*outer*_ were −1.1*%* for Ph1 and +0.1*%* for Ph2; see Fig. 2A. Absolute differences remained around ±0.02 MBq mL ^−1^ for all *AC*_*ref*_ measured for both phantoms, suggesting an adequate calibration of the DPC-PET used in this study for ^90^*Y* imaging at these activities. Relative percentage differences between *D*_*mean*_ values ($\overline {D}_{pet}^{VSV}$ or $\overline {D}_{pet}^{LDM}$ to $\overline {D}_{ref}^{MC}$) using *VOI*_*outer*_ for Ph1 were −2.6*%* for both VSV convolution and LDM, and for Ph2 were −4.5*%* for VSV convolution and −1.1*%* for LDM; see Table 3. However, differences were obtained in calculations of absorbed dose distributions comparing $DVH_{pet}^{VSV}$ and $DVH_{pet}^{LDM}$ to $DVH_{ref}^{MC}$, as shown in Fig. 2B and C for the two phantoms, illustrating the loss of accuracy brought by the use of the PET images compared to the ideal reference Monte Carlo simulations. The two figures also illustrate the PVEs on distributions of intermediate absorbed doses when using *VOI*_*outer*_ (the exact CT dimensions of the considered phantom VOIs) and *VOI*_*inner*_ (the reduced dimensions). Adding to PVE, there are also the low statistics and noise conditions in which imaging was performed, which could cause heterogeneity in the activity distribution and therefore in the absorbed dose distribution.

**Ph3 and evaluation through DVH.** The differences in $DVH_{ref}^{MC}$ and $DVH_{pet}^{VSV}$ are also depicted in Figs. 3, 4 and 5. The limitation due to the intrinsic poor statistics and the PVE compromise the accurate quantification of small objects, showing $DVH_{pet}^{VSV}$ and RMSDs which have large variations depending on the reconstruction parameters used. Siman et al. also showed relative large RMSDs between their reference and PET image-based DVHs, illustrating the loss of accuracy brought by the use of the images. Figure 5E and F depict the limit of reducing acquisition duration for small lesions. RMSDs between a 10 to a 15 min/bed acquisitions were significant for the 10- and 13-mm spheres compared to the other 4 larger spheres. On the other hand, acquisition duration can be reduced to 10 min/bed using a DPC-PET if the size of the lesion is at least 17 mm in diameter based on the obtained absorbed dose distributions in this study. The results found that in both the phantom and patient data, the difference in $DVH_{pet}^{VSV}$ between 10- and 15-min acquisitions was small, as depicted in Fig. 7.

**Variation of OSEM parameters.** The evaluation of the reconstruction parameters for dosimetry is necessary for each system, first owing to different PET performances in terms of sensitivity, spatial resolution, counts rates, energy and timing resolutions [30, 32, 35, 74, 75]; second, for different reconstruction algorithms, e.g. the OSEM or Bayesian Penalised Likelihood (BPL), where the implementations of OSEM-based algorithms vary from one manufacturer to another; and third, due to the very specific configurations of imaging protocols from one hospital to another.

The combination of parameters for OSEM reconstructions is not a simple choice and is specific for one configuration of SBR, *AC*_*ref*_, image voxel size and lesion size. In this study, the variation of iterations, limited from 1 to 3, did not have a significant impact on the calculated absorbed dose distributions; see Fig. 3. On the other hand, varying subsets and the FWHM of the post-reconstruction Gaussian filter had an impact. The use of 30 subsets could help in improving accuracy in dosimetry for the small spheres, but could favour noise amplification in the image compared to 10 subsets. The number of updates, which is the product of the number of iterations and subsets was not used as objective criteria for evaluation since different combinations for the same number of updates could provide different results, e.g. 30 updates for both i1s30-0mm and i3s10-0mm in Fig. 3.

**DVH comparisons using RMSD.** We evaluated the reconstruction parameters using RMSD comparisons between DVHs as suggested by Siman et al., but this could not be a relevant criteria for assessing absorbed dose distributions. Variations in RMSD will be observed depending on the range chosen for calculation, e.g. *D*_0*%*_−*D*_100*%*_,*D*_10*%*_−*D*_90*%*_ and *D*_20*%*_−*D*_80*%*_, as explained by Siman et al. themselves. In this study, whole range (*D*_0*%*_−*D*_100*%*_) was chosen for evaluation, even if larger RMSD would be obtained to include all factors which could affect the dosimetry.

It was found that the use of DVH is necessary and sufficient to make a choice on the reconstruction parameters. However, it depends on the information required (*D*_*mean*_,*D*_*max*_,*D*_20*%*_,*D*_50*%*_,*D*_80*%*_, etc.), and the size of the VOI. For example, for the 22–37-mm spheres in Fig. 3, a compromise can be made between intermediate absorbed doses, e.g. between *D*_20*%*_ and *D*_80*%*_, and the *D*_*max*_, where i3s10-0mm (or i3s10-2mm) can be suitable for reconstruction.

***RC***_***AC***_** and*RC***_***Dose***_**.** Owing to the few statistical production of positrons during ^90^Y decay, PVE and other confounding factors, the *RC*_*AC*_ does not reach 100% for any of the spheres in Ph3, as it can be the case for ^18^F imaging. This is true for all 69 PET systems evaluated in the QUEST multicentric study [45] in 2014, for any kind of reconstruction. No SiPM systems were included in the QUEST evaluation, due to SiPM PET systems only being commercialised from 2013 for Philips, 2016 for GE (Discovery™ MI) and 2018 (Biograph Vision™) and 2020 (Biograph Vision Quadra™) for Siemens. Since activity recovery has not reached at 100% for spheres up to 37 mm, absorbed doses for such lesion sizes are expected to suffer from poor quantitative accuracy and corrections in the absorbed dose estimations still need to be investigated and accounted for post ^90^Y-SIRT dosimetry. For the largest 28- and 37-mm spheres, the *RC*_*Dose*_ were around 0.8, and the $\overline {D}^{VSV}_{pet}$ seems to be underestimated by about roughly 20% if we compare to $\overline {D}^{MC}_{ref}$. The two smallest 10- and 13-mm spheres have underestimations on the mean absorbed dose which can be greater than 50%. The latter still suffer from greater PVE due to the spatial resolution, which is around 4-mm FWHM [29, 30, 32]. In an attempt to compare to ^18^F imaging but for qualitative studies and diagnostic purposes, Salvadori et al. [39] obtained CRCs which were less than 50% using 1 to 3 OSEM iterations for the 10-mm sphere on the DPC-PET, showing the limits of small spheres even for high *β*^+^ production statistics.

**Absorbed dose calculations.** VSV convolution for absorbed dose calculation is based on pre-calculated kernels by Monte Carlo methods and has been validated and proved to be clinically suitable for ^90^Y post-SIRT dosimetry [69]. LDM is a fast voxel-based method and easy to apply in clinic, which requires no post-processing and where a multiplicative factor similar to Eq. 1 is applied in a voxel-wise manner, as opposed to a pre-calculated convolution kernel for VSV. LDM is an alternative providing good accuracy as suggested by Pasciak et al. [37]. Monte Carlo simulations were used in this study but did not aim at replacing clinical dosimetry using VSV convolution or LDM. It was used as a tool to obtain a reference in absorbed dose distributions and was easier to use than industrial software for batch processing. It has not been detailed here, but absorbed dose distributions using VSV convolution were compared to PET image-based Monte Carlo simulations where excellent agreements were obtained between them, again illustrating the major image degradation coming from the non-ideal PET performance impacting on the absorbed dose distributions.

**Comparison of VSV convolution to LDM.** LDM would probably be a good method for absorbed dose computation, as shown by Pasciak et al. [37], since the PET derived ^90^Y absorbed dose distribution is already blurred by PVE and organ movement due to respiration, and indeed, there might be no need to blur even more the PET signal with a kernel. Results in this study show comparable $\overline {D}^{VSV}_{pet}$ and $\overline {D}^{LDM}_{pet}$ for phantoms and patients; see Tables 3, 4 and 5. However, LDM has the tendency to favour amplification of the *D*_*max*_ as seen on Fig. 2B and C (green DVHs), which adds a bias on the *D*_*mean*_ by increasing its value. In fact, considering Table 4, the $\overline {D}^{LDM}_{pet}$ is closer than $\overline {D}^{VSV}_{pet}$ to $\overline {D}^{MC}_{ref}$ for all spheres. If we consider Table 5, *D*_2*%*_ for LDM is 20 to 40 Gy higher than VSV convolution for different VOIs in patients. The same observations can be made using reference phantom data.

LDM seems to favour amplification of high absorbed doses compared to VSV convolution if compared to Monte Carlo simulations, to the detriment of intermediate absorbed doses. Therefore, this study suggests that considering the *D*_*mean*_ would not be the best criteria to assess absorbed doses using LDM. Finally, LDM and VSV convolution methods are both available for clinical practice. Even though more difficult to implement than LDM, VSV convolution is also fast (approx. 30 s for calculation per reconstructed image) and clinically feasible for each patient and both can be used if DVH comparisons are performed.

**Comparison to previous studies.** Following the improvements in photon detection in PET systems, PET/CT is an established and recommended method for ^90^Y treatment verification after SIRT as it provides improved accuracy for dosimetry [76]. A number of phantom studies have been performed with ^90^Y on different PET systems [23, 36–38, 45, 49–62]. They are summarised in Table 6. Some studies focused on qualitative and detection performances through image quality reports [36, 53, 55, 56, 58, 60–62], such as the CRC and the background variation (BV) following the NEMA NU-2 standards and guidelines [35]. Some other studies focused on a more quantitative evaluation on activity concentrations using *RC*_*AC*_ [23, 36, 38, 45, 49–52, 54, 57, 59, 60, 62]. Fewer phantom studies focused on improving dosimetric quantification using *RC*_*Dose*_ or other dosimetric clinical routine metrics [37, 38, 53, 57, 62]. Elschot et al. [53] in 2013 showed through DVH that ^90^Y dosimetry is more accurate for PET than SPECT imaging. Strydhorst et al. [56] in 2016 showed in their study that the bremsstrahlung radiation had negligible effects on PET-image image quality using Monte Carlo simulations. D’Arienzo et al. [57] in 2017 concluded that the post-SIRT dosimetry is possible even in conditions of low statistics and high random fraction, provided that accurate PET calibration is performed and acquisition durations are sufficiently long. Pasciak et al. [37] in 2014, Siman et al. [38] in 2018 and the latest study in 2020 from Hou et al. [62] were the only studies which suggested optimised reconstruction algorithm parameters using either, or both, *RC*_*Dose*_ and DVH estimations on phantoms. However, the suggested OSEM parameters from Pasciak et al. [37] (i1s21-0mm + 4.5 mm FWHM PSF + ToF) were different from Siman et al. [38] (i3s12-5.2mm + PSF + ToF) and were for different PET systems. Hou et al. [62] evaluated reconstructions on GE systems using a Penalised Likelihood (PL) algorithm.

**Table 6:** Summary of phantom studies with hot spheres for ^90^Y, for several PET/CT and PET/MR systems, SBR and *AC*_*ref*_

**Ref.**	**Scanner**	Spheres phantom set					
		SBR	*AC*_*ref*_	Contrast	Activity	Dose	Optim.
			(MBq mL ^−1^)				(Variable parameters)
Werner et al. [49]	Biograph Hi-Rez 16	N/A	3.6	-	x	-	-
Van Elmbt et al. [50]	Gemini TF						
	Gemini Power 16	3:1	1.3	-	x	-	-
	Ecat Exact HR+						
Bagni et al. [51]	Discovery ST	10:1	1.92	-	x	-	-
D’Arienzo et al. [52]							
Willowson et al. [36]	Biograph mCT-S(64)	8:1	3.9	x	x	-	OSEM
							(i1, i2, i3,
							s14, s21, s24)
Elschot et al. [53]	Biograph mCT	1:0	2.4	x	-	x	-
		9:1					
Carlier et al. [23]	Biograph mCT 40	40:1	8.1	-	x	-	OSEM
							(i1, i3)
Attarwala et al. [54]	Biograph mCT 40	8:1	2.38	-	x	-	OSEM
							(i1 to i12)
Martí-Climent et al. [55]	Biograph mCT-TrueV	5:1	1	x	-	-	OSEM
							(i1, i2, i3,
							2mm, 4mm, 6mm)
Pasciak et al. [37]	Biograph mCT Flow	3:1	2.2	-	-	x	PSF at FWHM (mm)
							(2 to 12)
Willowson et al. [45]	Various ^∗^	8:1	N/A	-	x	-	-
(The QUEST study)							
Strydhorst et al. [56]	Biograph mCT	8:1	N/A	x	-	-	-
D’Arienzo et al. [57]	Discovery ST	8:1	2.28	-	x	x	-
Siman et al. [38]	Discovery 690	4:1	1.6	-	x	x	OSEM
		13:1	4.8				(i1 to i12,
							0mm, 2.6mm, 5.2mm, 7.8mm, 10.4mm)
Maughan et al. [59]	Biograph mMR ^∗∗^	8:1	N/A	-	x	-	-
(The MR-QUEST study)							
Scott and McGowan [60]	Discovery 710	8:1	N/A	x	x	-	PL
Rowley et al. [58]	Discovery 710	8:1	3.3	x	-	-	PL
Seo et al. [61]	SIGNA (PET/MR)	4:1	N/A	x	-	-	-
Hou et al. [62]	Discovery 690	7.5:1	2.45	x	x	x	PL
The present study	Vereos DPC	9:1	2.25	-	x	x	OSEM
							(i1, i2, i3,
							s10, s20, s30,
							0mm, 2mm, 4mm, 6mm, 8mm
							5, 10, 15 min/bed)

The four last columns to the right summarise the type of evaluation done in the different studies. **Contrast**: Qualitative evaluation using definitions such as in the NEMA NU-2 standards for image quality. **Activity**: Quantitative evaluation either based on activity or *RC*_*AC*_ estimates. **Dose**: Quantitative evaluation based on *RC*_*Dose*_ or DVH estimates. **Optim**: Studies which aimed at varying reconstruction parameters to find optimised reconstruction parameters. Only variable parameters for OSEM reconstructions with ToF are reported

^∗^The QUEST phantom study including 69 PET/CT systems (GE, Siemens, Philips)

^∗∗^ The MR-QUEST phantom study including 8 PET/MR systems (Siemens)

**Limitations.** In the present study, we evaluated several $DVH_{pet}^{VSV}$ for the acquisition in specific conditions (SBR of 9:1, isotropic image voxel size of 2 mm, *AC*_*ref*_ of 2.18 MBq mL ^−1^). For further investigation, evaluations of $DVH_{pet}^{VSV}$ should be made for the different *AC*_*ref*_ present in the spheres at different imaging times and also by varying the image resolution, for example for voxels of 4 mm instead of 2 mm. Evaluations varying SBR would require more experimental data, with a different experimental setup for each SBR. Evaluations by varying and tuning the parameters chosen for the regularised version of the Richardson-Lucy algorithm for resolution recovery (fixed to 1 iteration with a 6 mm regularisation kernel in this study according to recommendations) and their influence on the accuracy of quantitative recovered information in the reconstructed images can be the topic for future studies. Selected reconstruction parameters were based on evaluations using VSV convolution, but similar evaluations can be performed using LDM. The results presented here can be useful in the choice of OSEM reconstruction parameters for example in studies such as published by Wei et al. [77], Levillain et al. [78], Morán et al. [79] and Hess et al. [80] for better accuracy in absorbed dose calculation following ^90^Y-SIRT using the DPC-PET.

## Conclusion

This study aimed to evaluate various parameters for ^90^Y-PET imaging with a DPC-PET Philips system for post-SIRT image-based dosimetry. To our knowledge, no previous study concerning the evaluation of acquisition and reconstruction parameters through DVHs have been published previously for SiPM PET systems. Overall, for dosimetry purposes, we recommend to apply a 2-mm FWHM post-reconstruction Gaussian filter size, which could reduce noise in the reconstructed image while keeping the same accuracy as when no filter is applied. The selected reconstruction parameter set could be i3s10-2mm for large spheres, but this choice depends on the absorbed dose information required. This study can be useful in the choice of reconstruction parameters using the DPC-PET, depending on imaging conditions for ^90^Y. The acquisition length can also be reduced from 15 to 10 min/bed for ^90^Y-SIRT with acceptable accuracy degradation in the absorbed dose distribution, improving patient comfort.

## Data Availability

GATE scripts for simulation during the current study are available from the corresponding author on request.

## Footnotes

1 See https://geant4.web.cern.ch/node/1731
